# Disappearance of a Ruptured Feeding Artery Fusiform Aneurysm After the Resection of a Large Subependymoma: A Case Report

**DOI:** 10.7759/cureus.48873

**Published:** 2023-11-15

**Authors:** Akinari Yamano, Masahide Matsuda, Hisayuki Hosoo, Eiichi Ishikawa

**Affiliations:** 1 Department of Neurosurgery, Institute of Medicine, University of Tsukuba, Tsukuba, JPN

**Keywords:** hemodynamic stress, vessel wall stress, fusiform aneurysm, flow-related aneurysm, intracerebral hemorrhage, feeding artery, aneurysm, intraventricular tumor, subependymoma

## Abstract

Aneurysm formation on the tumor-feeding artery is rare, and its treatment strategies are not yet settled. We herein report the case of a 49-year-old female with a large subependymoma in the left lateral ventricle presenting remote intracerebral hemorrhage at the left posterior cingulate gyrus. Digital subtraction angiography (DSA) revealed the presence of a 5.5 mm fusiform tumor-feeding artery aneurysm on the left parieto-occipital branch of the posterior cerebral artery, considered to be the source of the hemorrhage. Three months after total tumor resection, the aneurysm subsequently disappeared on the follow-up angiography. Subependymomas are generally known as tumors with low vascularity and seldom present with symptoms such as intracerebral hemorrhage. From the subsequent disappearance of the aneurysm after the complete tumor resection, the pathophysiological cause of the aneurysm formation is assumed to be flow-related hemodynamic vessel wall stress of the feeding artery. Tumor resection alone may be a favorable first treatment strategy to avoid unnecessary brain damage since subsequent disappearance of the aneurysm can be expected. The coexistence of feeding artery aneurysms should be kept in mind, especially in cases with remote hemorrhage.

## Introduction

Brain tumors sometimes have coexisting intracranial aneurysms [1,2], but because such aneurysm formation on the tumor-feeding artery is rare [3-5], treatment strategies are not yet settled [6]. Herein, we present a case of a giant subependymoma in the lateral ventricle with a ruptured feeding artery fusiform aneurysm that angiographically disappeared after the total resection of the tumor. This is the first report of a subependymoma with tumor-feeding artery ruptured aneurysm and subsequent aneurysm disappearance after tumor resection. This report discusses the pathophysiological cause of and treatment strategy for coexisting aneurysms.

## Case presentation

**Figure 1:**
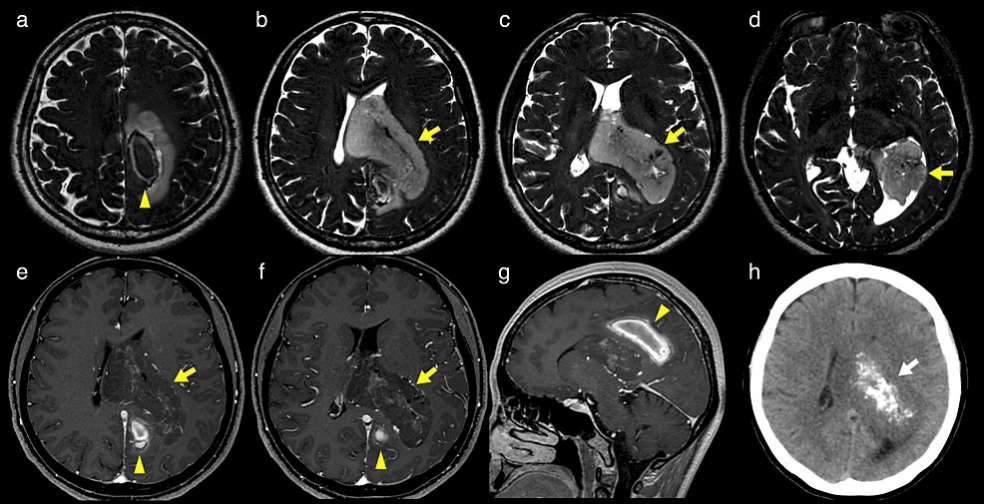
Magnetic resonance imaging and computed tomography Magnetic resonance imaging reveals a T2 hyperintense intraventricular mass with wispy contrast enhancement in the left lateral ventricle (yellow arrow) and T1 hyperintense and T2 hypointense intracerebral hemorrhage at the left cingulate gyrus, which is separated from the intraventricular mass (yellow arrowhead). (a-d) Axial T2 DRIVE images. (e,f) Axial T1-weighted images with gadolinium. (g) Sagittal T1-weighted image with gadolinium. (h) Axial computed tomography image shows intra-tumoral calcification (white arrow).

**Figure 2:**
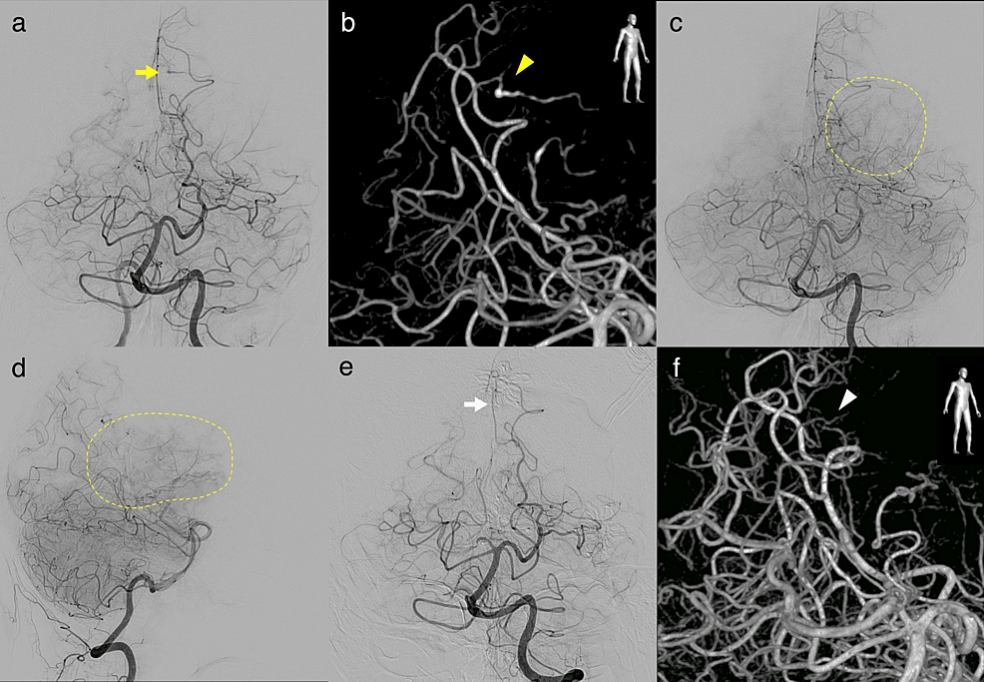
Preoperative and postoperative DSA (a) Preoperative DSA from the left vertebral artery reveals a fusiform aneurysm (yellow arrow) on the left parieto-occipital artery, which is a feeding artery of the intraventricular mass. (b) 3D-RA shows the location of the aneurysm (yellow arrowhead). (c,d) Preoperative DSA shows a faint tumor stain in the capillary phase, indicating low vascularity of the tumor (surrounded by the dotted line). (e) Postoperative DSA, performed three months after the second surgery, indicates the regression of the aneurysm (white arrow). (f) Postoperative 3D-RA shows slight flow in the artery where the aneurysm was present (white arrowhead). DSA: digital subtraction angiogram, 3D-RA: three-dimensional rotational angiogram

A 49-year-old female with no notable previous medical history presented with sudden dysmnesia and right sensory disturbance. The patient had not experienced any noticeable symptoms before the onset. Physical examination showed no physical function deficit. However, the patient had short-term memory and working memory disturbance. The Mini-Mental State Examination (MMSE) score was recorded at 27 points. Magnetic resonance imaging (MRI) revealed an 8 cm mass in the left lateral ventricle and an intracerebral hemorrhage at the left posterior cingulate gyrus (Figure 1a-1d). The intraventricular mass extended from the anterior horn to the inferior horn. Digital subtraction angiography (DSA) showed a 5.5 mm fusiform aneurysm on the parieto-occipital branch of the posterior cerebral artery, which was assumed to be the source of the hemorrhage (Figure 2a, 2b). Based on the faint tumor stain in the DSA, the vascularity of the tumor was considered low (Figure 2c, 2d).

We performed a two-stage tumor resection because of the tumor size and location. A transcortical approach from the left superior frontal gyrus was taken as the first stage, with a transcortical approach from the left superior parietal lobule as the second stage after a one-month interval. We only removed the tumor, and cingulate gyrus hematoma was not observed during the two surgeries. Gross total resection of the tumor was achieved, and the pathological diagnosis was subependymoma, World Health Organization grade 1 [7]. Although the cingulate gyrus hematoma and fusiform aneurysm were not treated during the surgical procedure, the aneurysm was not observed in the DSA performed three months post-surgery (Figure 2e, 2f). The patient had transient cognitive impairment after the surgery, which subsequently improved. MRI has shown no signs of recurrence for six months.

## Discussion

The coexistence of primary brain tumors and intracranial aneurysms is reported at an incidence of 1.1%-5.4% [1,2], but most of these aneurysms are located at common sites such as the internal carotid, anterior communicating, and middle cerebral arteries [6]. While distal flow-related aneurysms on vessels directly supplying arteriovenous malformations (AVMs) are frequently reported, an aneurysm on a tumor-feeding artery is extremely rare [8]. The few published cases of feeding artery aneurysms associated with intra-axial tumors are reported mostly in patients with hypervascular tumors such as glioblastomas and hemangioblastomas [3,4]. There is only one report of a hypovascular tumor with a flow-related aneurysm on the feeding artery, and the pathology was central neurocytoma [5]. Subependymomas are rare intracranial tumors, mostly benign with low vascularity [9,10], that seldom appear as symptomatic (usually by obstructive hydrocephalus or intracranial hemorrhage) [11,12]. As far as we are aware, this is the first case of a subependymoma presenting with remote intracerebral hemorrhaging. The pathophysiological cause of the aneurysm formation remains uncertain, but hemodynamic vessel wall stress is assumed to be involved in our case.

Hemodynamic vessel wall stress is considered a primary cause of flow-related aneurysms in AVMs. Notably, distal flow-related aneurysms are assumed to have a strong association with vessel wall stress and a higher probability of regression after removal of the nidus [8]. In our case, the aneurysm was located on the artery that directly fed the tumor, resembling distal flow-related aneurysms in AVMs. The subsequent disappearance of the aneurysm after complete tumor resection suggests its flow-related nature and the involvement of hemodynamic stress. However, the fact that subependymomas are hypovascular tumors does not completely fit with the flow-related hypothesis. It is possible that the large tumor size may result in rich blood flow, and there is one reported case of a tumor with low vascularity presenting a similarly flow-related aneurysm [5]. As another hypothesis, the vascular fragility of the tumor-feeding artery may have contributed to aneurysm formation. In this regard, possible factors could include the tumor invasion of the arterial wall [13], hormone secretion from the tumor [14], or other signaling molecules released by the tumor [15]; however, none of these theories are consistent with the present case. Although the lack of aneurysmal tissue samples makes it unclear what other factors were involved in aneurysm formation, we consider the main cause to be flow-related hemodynamic vessel stress.

There is no unified view about treatment strategies for brain tumor-feeding artery aneurysms [6]. Notably, the aneurysm was symptomatic, but the hematoma was unattached to the tumor in our case. To directly treat the fusiform aneurysm, both endovascular and surgical trapping of the artery are feasible, but endovascular trapping has a risk of ischemic complications in the distal territory. Surgical trapping carries the same ischemic risk but additionally requires a new surgical tract via the brain parenchyma. Since the feeding artery was narrow and the blood flow was not so intense, we assumed that the immediate risk of re-rupture was low. As distal flow-related aneurysms have a high rate of regression after the AVM treatment [8], we decided to remove the tumor first and monitor for subsequent disappearance of the aneurysm. DSA three months after surgery showed complete regression of the aneurysm, and no hemorrhagic complications were present. In this type of flow-related aneurysm on the feeding artery, a good clinical choice may be to remove only the tumor first as a treatment strategy to prevent further brain damage.

## Conclusions

Brain tumors with coexisting tumor-feeding artery aneurysms are rare. However, even if brain tumors have low vascularity, the coexistence of feeding artery aneurysms should be kept in mind, especially in cases with remote hemorrhage. Subsequent regression of the aneurysm may be expected with tumor removal, so aneurysm trapping may be proscribed if the immediate re-bleeding risk is considered to be low.
